# A comparative study on the anti-inflammatory effects of single oral doses of naproxen and its hydrogen sulfide (H_2_S)-releasing derivative ATB-346 in rats with carrageenan-induced synovitis

**DOI:** 10.1186/2045-9912-3-24

**Published:** 2013-11-16

**Authors:** Eduardo Ekundi-Valentim, Filiphe PN Mesquita, Karen T Santos, Marco A Vieira de Paula, Juliana Florenzano, Cristiane I Zanoni, Leandro Rodrigues, Gilberto de Nucci, Simone A Teixeira, Heloisa HA Ferreira, John L Wallace, Soraia KP Costa, Marcelo N Muscará

**Affiliations:** 1 Polytechnic Institute of Malanje, Lueji A'Nkonde University, Malanje, Angola; 2 Department of Pharmacology, Institute of Biomedical Sciences, University of Sao Paulo, Av. Prof. Lineu Prestes, 1524, São Paulo 05508-000, SP, Brazil; 3 Laboratory of Inflammation Research, Sao Francisco University, Bragança Paulista, SP, Brazil; 4 Farncombe Family Digestive Health Research Institute, McMaster University, Hamilton, ON, Canada

**Keywords:** Hydrogen sulfide, NSAID, ATB-346, Synovitis, Joint, Inflammation, Rat

## Abstract

**Background:**

Non-steroidal antiinflammatory drugs (NSAIDs) are the most commonly prescribed agents for arthritic patients, although gastric effects limit their long-term use. Considering the reported gastric safety of hydrogen sulfide (H_2_S)-releasing NSAIDs, in addition to the anti-inflammatory effects of H_2_S administration to rats with synovitis, we decided to evaluate the effects of the H_2_S-releasing naproxen derivative ATB-346 in this animal model.

**Methods:**

Male Wistar rats were anesthetized with inhalatory halothane and pre-treated with equimolar oral doses of either naproxen (0.3, 1, 3 or 10 mg/kg) or ATB-346 (0.48, 1.6, 4.8, or 16 mg/kg) 30 min before the i.art. injection of 7.5 mg of carrageenan (CGN) into the right knee joint cavity. Joint swelling and pain score were assessed after 1, 3 and 5 h, and tactile allodynia after 2 and 4 h. After the last measurement, the joint cavity lavages were performed for counting of the recruited leukocytes. The drugs (at the highest doses) were also tested for their gastric effects by evaluating macroscopical damage score and neutrophil recruitment (measured as myeloperoxidase – MPO activity) in the stomachs 5 h after administration of the drugs. In addition, the serum naproxen pharmacokinetic profiles of both compounds, administered at the highest equimolar doses, were obtained during the first 6 h after dosing.

**Results:**

At the two highest tested doses, both naproxen and ATB-346 reduced edema and pain score (measured 3 and 5 h after CGN; P < 0.001). Tactile allodynia was similarly inhibited by ~45% 4 h after CGN by both naproxen (at 1, 3 and 10 mg/kg) and ATB-346 (at 1.6 and 4.8 mg/kg; P < 0.001), as well as leukocyte infiltration. Naproxen (but not ATB-346) induced significant gastric damage and, despite the increased gastric MPO activity by ~130% in the naproxen-, but not in the ATB-346-treated rats, this effect was of no statistical significance.

**Conclusion:**

The presence of a H_2_S-releasing moiety in the ATB-346 structure does not impair the antiinflammatory activity of the parent compound in rats with CGN-induced synovitis. In addition, released H_2_S may account for the absence of deleterious gastric effects, thus making of ATB-346 a potentially useful therapeutic alternative to traditional naproxen for treatment of patients with arthritis.

## Background

Rheumatoid arthritis (RA) is a chronic inflammatory autoimmune disease, and its pharmacological treatment just aims to alleviate the associated pain, to control inflammation, to preserve function and to prevent the consequent deformities. Both steroidal and non-steroidal anti-inflammatory (NSAIDs) drugs, and disease modifying antirheumatic agents are among the available therapeutic tools [1].

NSAIDs are the most widely used drugs for the relief of pain, swelling and stiffness of the joints in RA [2]; however, in addition to the higher risks of renal and cardiovascular occurrences [3], the chronic use of NSAIDs results in clinically significant gastrointestinal ulceration and bleeding [4]. The mechanisms underlying these gastric damage events include both direct toxic effects of the NSAIDs on the epithelial cells, and others related to the prostaglandin synthesis inhibition, such as the reduction of mucus and bicarbonate secretion [5] and the increased neutrophil adherence and activation [6]. In this way, attempts to design NSAIDs that do not cause gastrointestinal damage still face the challenge of overcoming the detrimental effects of suppression of prostaglandin synthesis while maintaining the beneficial effects of these drugs, which are also related to inhibition of the COX enzymes.

Over the last decade, knowledege on the physiological relevance of the gaseous mediator hydrogen sulfide (H_2_S) has significantly increased, evidencing the protective functions of this gasotransmitter in the gastrointestinal tract [7], the cardiovascular system [8], the central nervous system [9], and as an endogenous modulator of leukocyte adherence to vascular endothelium [10]. In addition, the anti-inflammatory properties of exogenously administered H_2_S have been observed in several experimental models such as carrageenan-induced paw edema in rats [10], myocardial ischemia-reperfusion injury in pigs [11], arthritis [12-14] and lung injury [15] and asthma [16] in mice.

Taking these facts together, novel H_2_S-releasing NSAID derivatives were developed, tested in several animal models of inflammation and, in many respects such as gastric effects, these compounds have shown significant advantages over the parent anti-inflammatory drugs (for review, see [17]).

In the present study, we compare naproxen with its H_2_S-releasing derivative ATB-346 [2-(6-methoxy- napthalen-2-yl)-propionic acid 4-thiocarbamoyl-phenyl ester] in terms of anti-inflammatory and analgesic effects when administered as single doses to rats with carrageenan-induced knee joint synovitis.

## Material and methods

### Animals

Wistar rats (180-200 g) from the local animal care facilities were used in this study. All the experimental procedures are in accordance with the ethical principles for animal research set down in the Animals (Scientific Procedures) Act, UK, 1986 and were approved by the local ethics committee at the University of Sao Paulo (protocol N° 64, book n° 2/2007). Rats were kept in polypropylene cages (5 per cage) under standard controlled conditions (22°C; 12 h light/dark cycle) with free access to commercial rodent chow and tap water.

### Experimental design

The rats were orally pre-treated with either vehicle (1 ml/kg of 0.5% carboxy methylcellulose; Cromoline Química Fina Ltda., Diadema, SP, Brazil), naproxen - NAP (0.30, 1.0, 3.0 or 10 mg/kg; Sigma-Aldrich Co. LLC, St. Louis, MO, USA), or the corresponding equimolar doses of the H_2_S-releasing naproxen derivative ATB-346 (0.48, 1.6, 4.8, or 16 mg/kg; Antibe Therapeutics Inc., Toronto, ON, Canada) 30 min before the i.art. injection of 7.5 mg of carrageenan (Sigma-Aldrich Co. LLC, St. Louis, MO, USA). Joint swelling (measured as the mediolateral knee diameter) and pain-related behaviour (analysed as gait score) were blindly assessed 1, 3 and 5 h after CGN, and secondary tactile allodynia (by means of an electronic Von-Frey-based device) was measured after 2 and 4 h, as previosuly described [13]. After the last end-point measurement, joint cavity lavages were collected for leukocyte counting [13].

The gastric effects of the treatments were also evaluated in additional groups of rats 5 h after receiving the highest naproxen or ATB-346 doses (10 and 16 mg/kg, respectively) by macroscopical blind examination of gastric damage [18] and neutrophil recruitment by measurement of myeloperoxidase (MPO) activity in the excised stomachs, as previously described [19].

### Measurement of plasma naproxen concentrations

In order to compare the oral bioavailability of naproxen from the tested compounds, groups of rats received equimolar doses of naproxen (10 mg/kg, p.o.) or ATB-346 (16 mg/kg, p.o. or i.v.), and at selected time points (up to 6 h after dosing), blood samples were collected from the descending abdominal aorta into EDTA containing tubes. Plasma was obtained by centrifugation of the tubes (2,000 *g* at 4°C during 10 min), and naproxen concentrations were measured by high-performance liquid chromatography coupled to electrospray tandem mass spectrometry (HPLC-MS-MS) using diclofenac as internal standard.

Chromatography was performed on a Genesis Lightn C8 4 μm analytical column (100 × 2.1 mm i.d.). The method had a chromatographic run time of 2.5 min and a linear calibration curve over the range 1-180 μg/ml (r^2^ > 0.9965; limit of quantification: 1 μg/ml).

To 100 μl aliquots of the plasma samples (or calibration standards) were sucessively added 50 μl of the internal standard solution (50 μg/ml diclofenac), 500 μl of HPLC-grade water, 20 μl of formic acid. After vortex mixing during 10 s, the compounds of interest were liquid-liquid extracted with 4 ml of diethyl ether-hexane (80:20, v/v) mixture and vortex mixed for 40 s; the upper organic layer was transferred to clean tubes and the solvent evaporated under a gentle N_2_ stream at 40°C. The dry residue was redissolved with 2 ml of mobile phase (a 80:20 v/v acetonitrile:water solution containing 1 mM acetic acid and 1 mM sodium acetate). The samples were transferred into glass microvials, capped, placed in an autosampler.

The LC10AD HPLC system (Shimadzu, Kyoto, Japan) consisted of a pump (operated at room temperature) and an autosampler (maintained at 7°C) set up to inject 10 μL. MS was performed in a Sciex API 3000 triple stage quadrupole MS (Applied Biosystems, Foster City, CA), equipped with an APPI source operating in negative mode using nitrogen as the collision gas. The ions were monitored in multiple reaction monitoring, and the transitions m/z 229.10 → 170.00 and m/z 296.10 → 251.90 were used for quantitation of naproxen and diclofenac (internal standard), respectively. Data were acquired with the Analyst software (version 1.3.1, Applied Biosystems, Cheshire, U.K.) and calibrations curves for the analyte were constructed using the naproxen-to-diclofenac peak-area ratio via a weighted (1/*x*^2^) least-squares linear regression. Unknown sample peak-area ratios were then interpolated from the calibration curve to obtain the naproxen concentration values.

### Statistical analysis

All results were expressed as mean ± standard error of the mean (SEM) for n animals. Differences among the group means were analysed by one-way ANOVA followed by the Bonferroni’s test for multiple comparisons. Gait score medians were analysed by the non-parametric Kruskal-Wallis test followed by the Dunn's test for multiple comparisons. For both types of statistical analysis, we used the software GraphPad Prism (version 4.0; GraphPad Software Corporation, San Diego, CA, USA). Values of P lower than 0.05 were considered as significant.

## Results

### Pain evaluation

**Figure 1:**
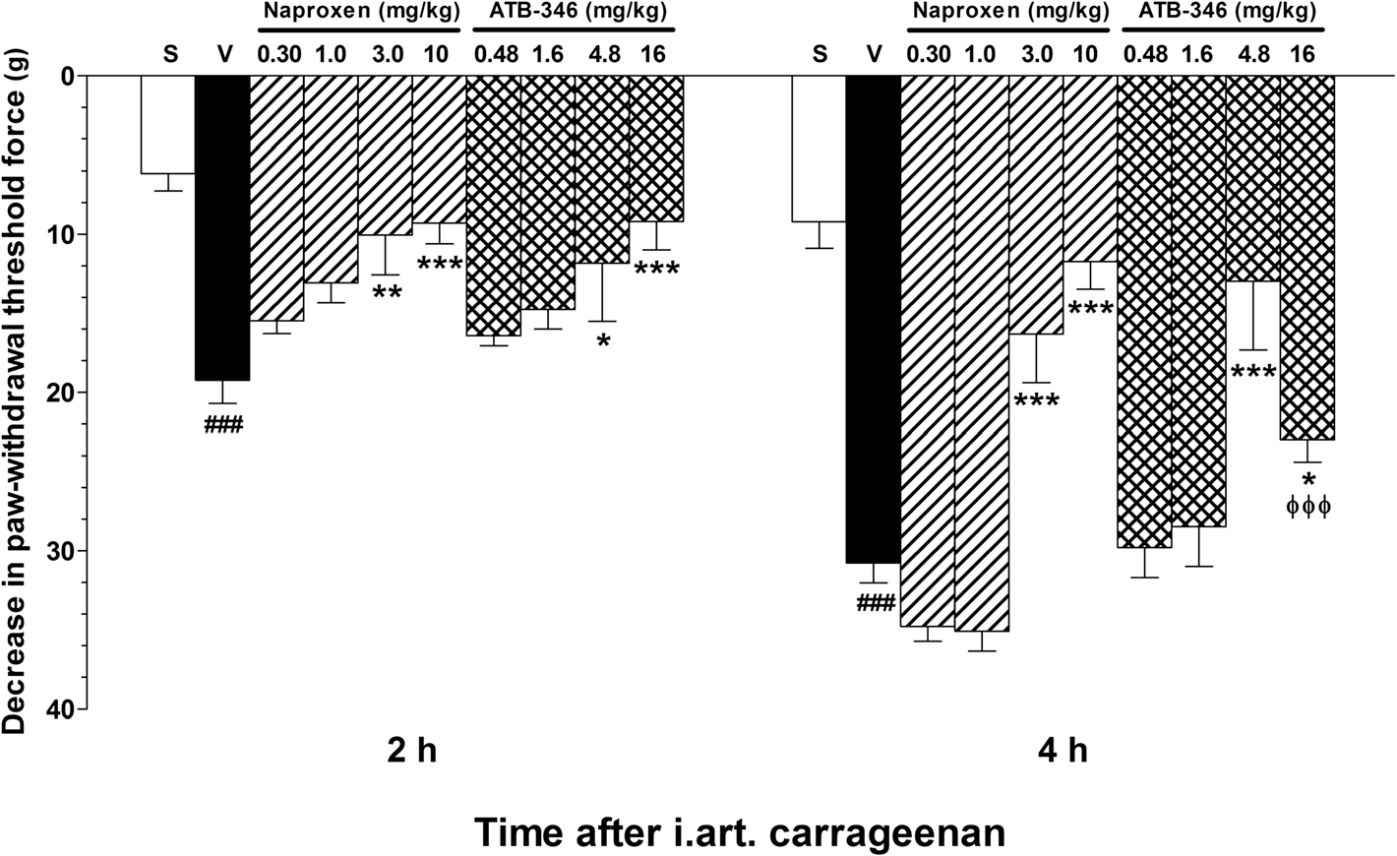
**Effects of treatment with single oral doses of naproxen or ATB-346 on tactile allodynia secondary to CGN-induced synovitis in rats.** CGN-injected animals were orally pre-treated with naproxen (at 0.3, 1, 3 and 10 mg/kg; n = 8), equimolar ATB-346 doses (0.48, 1.6, 4.8 and 16 mg/kg; n = 8) or vehicle (1 ml/kg; n = 8). A separate control group (Sham - S) received only an i.art. injection of saline injection (n = 5). The bars represent the mean ± SEM values of the changes in paw withdrawal force thresholds measured 2 and 4 h after CGN injection. *P < 0.05 and **P < 0.01 vs. vehicle-treated (V) group; ^###^P < 0.001 vs. S; ^ϕϕϕ^P < 0.001 vs. the corresponding equimolar naproxen dose, as analysed by one-way ANOVA followed by the Bonferroni's multiple comparison test.

As shown in Figure 1, the i.art. injection of carrageenan resulted in significant secondary tactile allodynia in the ipsilateral hindpaw in comparison with saline solution injection, and pre-treatment of the animals with either naproxen (3.0 and 10 mg/kg) or the equimolar ATB-346 doses (4.8 and 16 mg/kg, respectively) resulted in significantly reduced responses as evaluated 2 or 4 h after the carrageenan injection. Except for the lower effects observed in the 16 mg/kg ATB-346 group in comparison with the corresponding 10 mg/kg naproxen group (P < 0.001) 4 h after the carrageenan injection, no other significant differences were observed between naproxen and ATB-346 when administered at equimolar doses.

**Figure 2:**
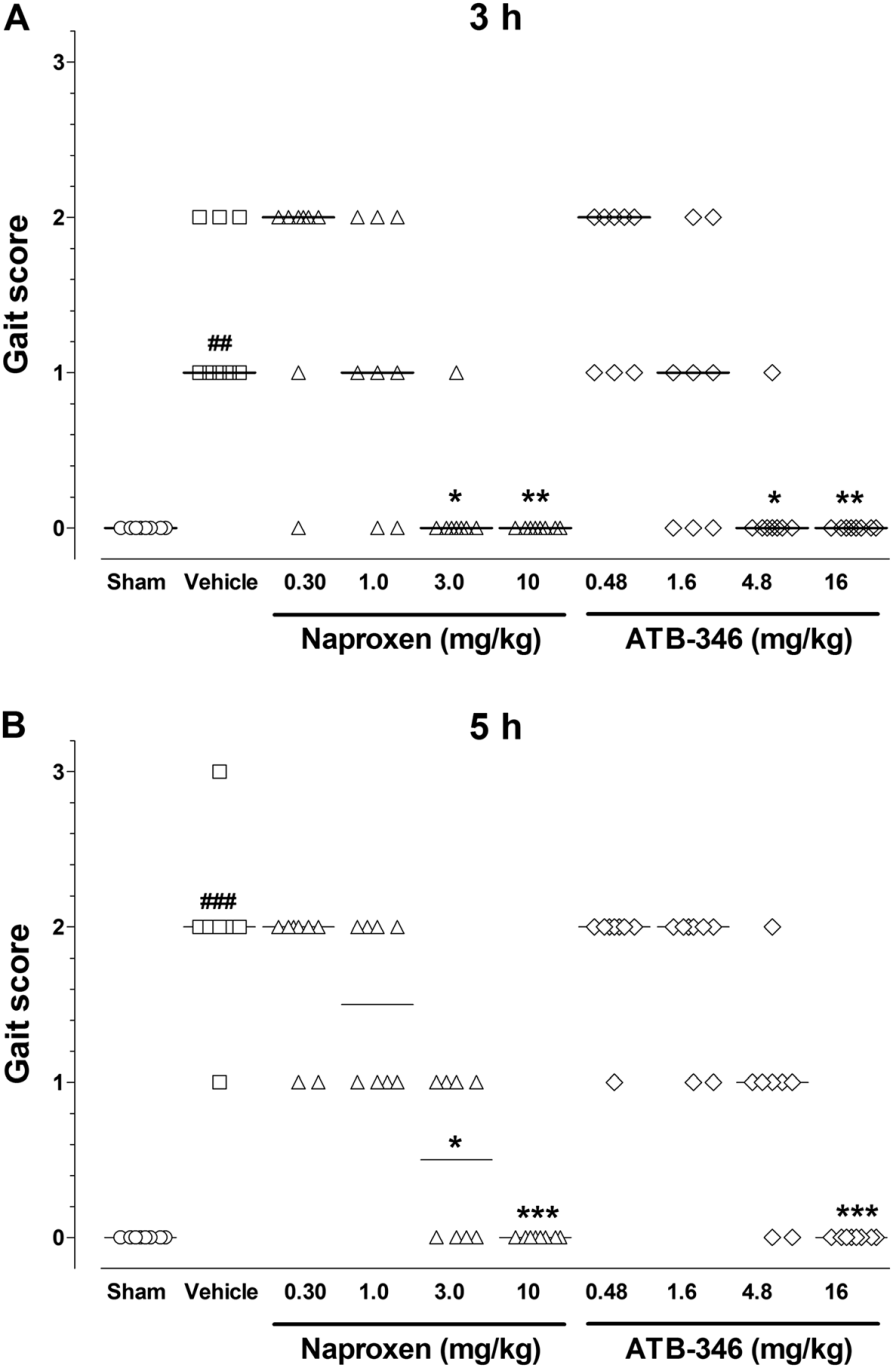
**Effects of treatment with single oral doses of naproxen or ATB-346 on gait score secondary to CGN-induced synovitis in rats.** CGN-injected animals were orally pre-treated with naproxen (at 0.3, 1, 3 and 10 mg/kg; n = 8), equimolar ATB-346 doses (0.48, 1.6, 4.8 and 16 mg/kg; n = 8) or vehicle (1 ml/kg; n = 8). A separate control group (Sham - S) received only an i.art. injection of saline injection (n = 5). Panels **A** and **B** illustrate the walking behaviours evaluated 3 and 5 h after CGN injection, respectively, which were scored on a scale from 0 (normal) to 3 (total joint immobility). Data are presented as scatter plots with the median values for each experimental group, and differences among the groups were non-parametrically analysed by the Kruskal-Wallis test followed by the Dunn’s test for multiple comparisons. *P < 0.05, **P < 0.01 and ***P < 0.001 vs. the vehicle-treated (V); ^##^P < 0.01 and ^###^P < 0.001 vs. the corresponding S group.

Similarly, the i.art. injection of carrageenan resulted in significant impairment of the normal walking pattern, as evidenced by the increased gait score observed after 3 or 5 h (Figure 2), and pre-treatment of the animals with either naproxen (3.0 and 10 mg/kg; n = 8) or ATB-346 (4.8 and 16 mg/kg) 30 min before carrageenan, resulted in significant decrease of this score, except for the 4.8 mg/kg ATB-346 treated group evaluated at the 5 h time-point.

### Articular edema

**Figure 3:**
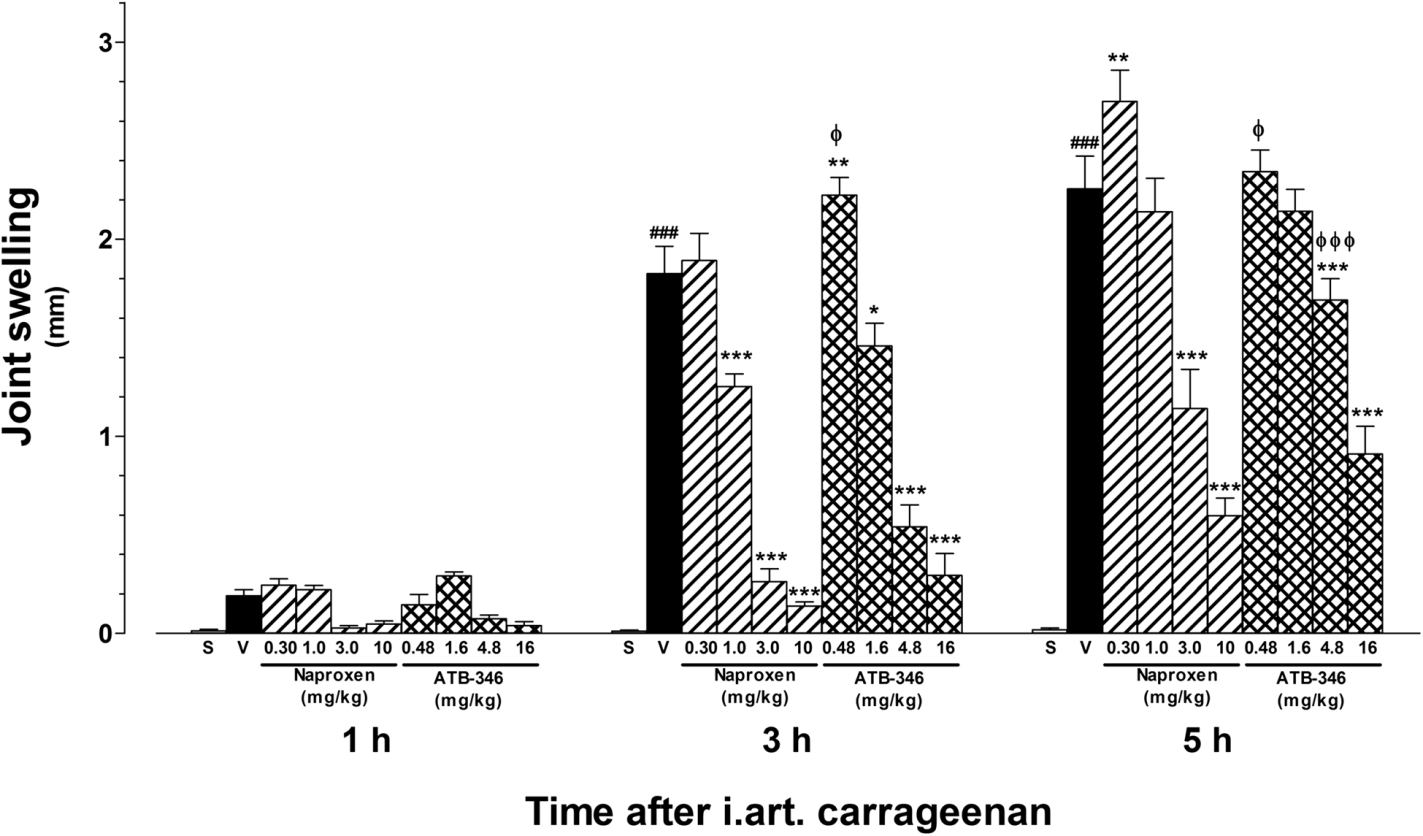
**Effects of treatment with single oral doses of naproxen or ATB-346 on knee joint swelling secondary to CGN-induced synovitis in rats.** CGN-injected animals were orally pre-treated with naproxen (at 0.3, 1, 3 and 10 mg/kg; n = 8), equimolar ATB-346 doses (0.48, 1.6, 4.8 and 16 mg/kg; n = 8) or vehicle (1 ml/kg; n = 8). A separate control group (Sham - S) received only an i.art. injection of saline injection (n = 5). The bars represent the mean ± SEM values of the increases in articular diameters (in mm) measured 1, 3 and 5 h after CGN injection. **P < 0.01 and ***P < 0.001 vs. vehicle-treated (V) group; ^###^P < 0.001 vs. S; ^ϕ^P < 0.05 and ^ϕϕϕ^P < 0.001 vs. the corresponding equimolar naproxen dose, as analysed by one-way ANOVA followed by the Bonferroni's multiple comparison test.

The injection of carrageenan resulted in significant increase in joint diameter, as measured after 3 or 5 h (but not after 1 h), in comparison with saline solution injection (Figure 3). At the 3 h time-point, pre-treatment with either naproxen (at 1.0, 3.0 or 10 mg/kg) or ATB-346 at the corresponding equimolar doses (1.6, 4.8 and 16 mg/kg, respectively) resulted in significantly reduced edema in comparison with vehicle. However, the animals pre-treated with 0.48 mg/kg ATB-346 showed a slightly higher, although statistically significant, articular edema than the animals pre-treated with either the vehicle or the equimolar naproxen dose (0.30 mg/kg). Five hours after the carrageenan injection, the knee joint edema was significantly reduced in the animals pre-treated with either naproxen (at 3.0 and 10 mg/kg) or ATB-346 at the equimolar doses, although the edema inhibition observed in the 4.8 mg/kg ATB-346-treated group was significantly weaker than that caused by the equimolar naproxen dose. In addition, pre-treatment with 0.30 mg/kg naproxen significantly potentiated the carrageenan-induced edema at this time-point.

### Leukocyte recruitment into the articular cavity

**Figure 4:**
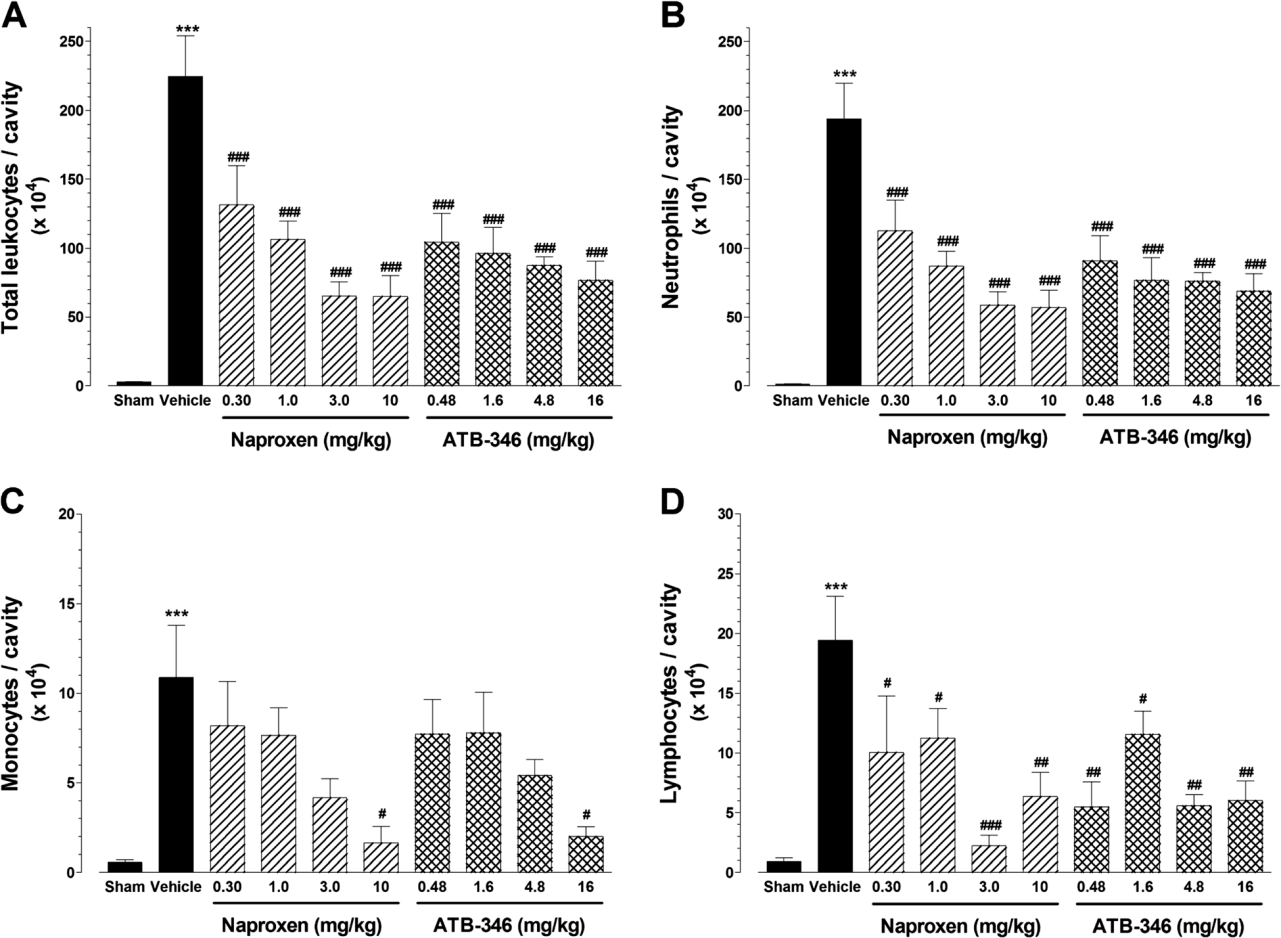
**Effects of treatment with single oral doses of naproxen or ATB-346 on leukocyte recruitment to the knee joint cavity secondary to CGN-induced synovitis in rats.** CGN-injected animals were orally pre-treated with naproxen (at 0.3, 1, 3 and 10 mg/kg; n = 8), equimolar ATB-346 doses (0.48, 1.6, 4.8 and 16 mg/kg; n = 8) or vehicle (1 ml/kg; n = 8). A separate control group (Sham - S) received only an i.art. injection of saline injection (n = 5). The bars represent the mean ± SEM values of the number of total leukocytes **(panel A)**, neutrophils **(panel B)**, monocytes **(panel C)** or lymphocytes **(panel D)** per cavity measured 5 h after CGN injection. ***P < 0.001 vs. S; ^#^P < 0.05, ^##^P < 0.01 and ^###^P < 0.001 vs. vehicle-treated (V) group, as analysed by one-way ANOVA followed by the Bonferroni's multiple comparison test.

As shown in Figure 4, the i.art. injection of carrageenan resulted in highly significant leukocyte recruitment to the joint cavity, as evidenced by the cell counts present in the synovial lavage fluid samples collected after 5 h. As it can be observed, neutrophils (panel B) account for most of the total leukocyte counts (panel A), although significant increases of both mononuclear cells (panel C) and lymphocytes (panel D) also ocurred in response to carrageenan. At all the tested doses, pre-treatment with either naproxen or ATB-346 resulted in significant reduction of total leukocytes, neutrophils and lymphocytes to comparable degrees between the corresponding equimolar doses of both compounds; however, only at the highest doses, both naproxen and ATB-346 were effective to significantly reduce the number of mononuclear cells in the carrageenan-injected joint cavities (panel C).

### Gastric effects

The blind macroscopical examination of the gastric tissues revealed that the samples obtained from rats treated with 16 mg/kg ATB-346 were indistinguishable from those from rats treated with the vehicle (i.e., score zero). In contrast, the administration of 10 mg/kg naproxen resulted in the development of gastric erosions in all 8 rats (damage score: 17 ± 3), which was significantly greater than those seen in the other groups (P < 0.001). In addition, the obtained gastric MPO activity results showed a similar profile among the groups (vehicle: 6.4 ± 2.8, naproxen: 14.4 ± 4.7 and ATB-346: 5.0 ± 1.2 U/mg of protein); however, these values were not statistically different when analysed by one-way ANOVA.

### Naproxen bioavailability

**Figure 5:**
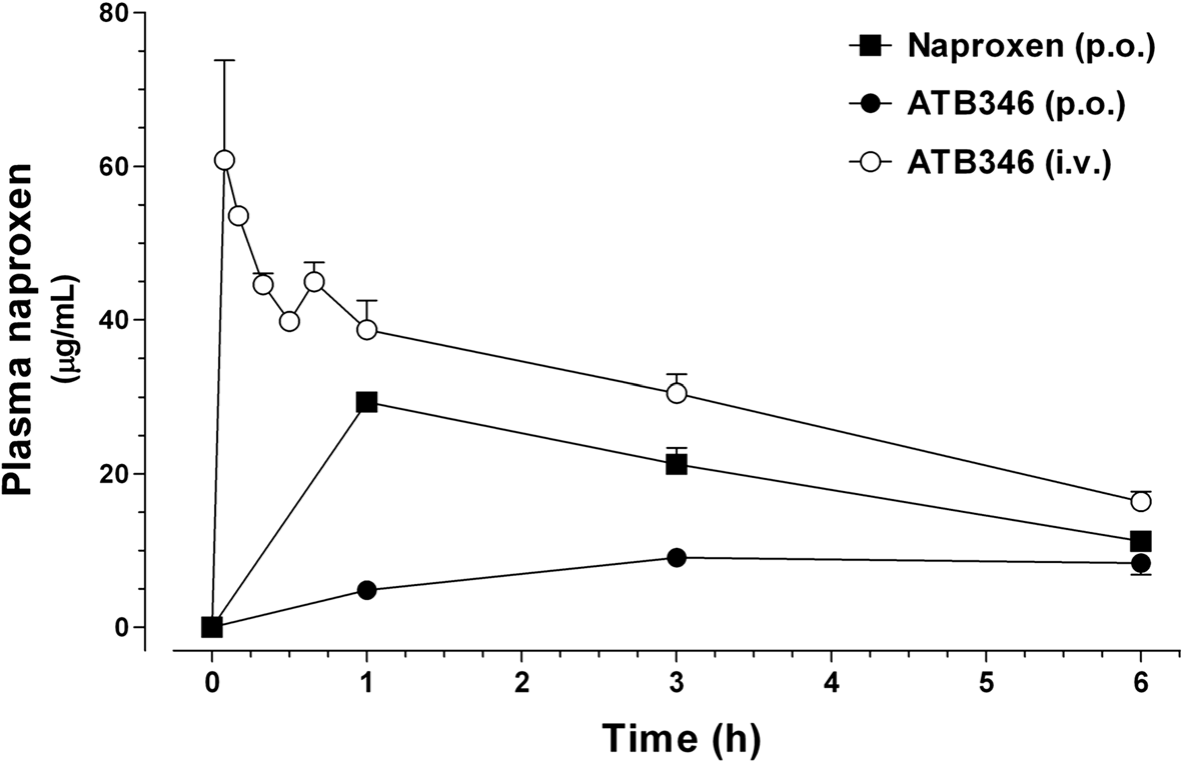
**Pharmacokinetic profiles of circulating naproxen following the administration of single doses of either naproxen or ATB-346 to normal rats.** Plasma naproxen concentration vs. time curves were obtained after the administration of equimolar doses of either naproxen (10 mg/kg; p.o) or ATB-346 (16 mg/kg; p.o and i.v) and measurement of the resulting naproxen concentrations by HPLC-MS-MS. Mean plasma concentrations (± SEM; n = 4) are expressed in μg/ml.

Figure 5 shows the kinetic profiles of plasma naproxen concentrations following the oral administration of 10 mg/kg naproxen and 16 mg/kg ATB-346 by both oral and intravenous routes along the first 6 h. Based on the mean values of the calculated areas under the curves, naproxen bioavailability from orally administered ATB-346 is approximately 23% of that observed following its i.v. administration (42.5 vs. 183.2 μg^.^h/ml, respectively), and approximately 37% of the naproxen bioavailability following the oral administration of the parent compound (113.9 μg^.^h/ml).

## Discussion

The present study shows that both naproxen and its H_2_S-releasing derivative ATB-346 exert acute anti-inflammatory and analgesic actions when administered as single oral doses to rats subjected to the CGN-induced knee joint synovitis. As a whole, the therapeutic profile of the compounds, as assessed by their inhibiting effects on joint swelling, inflammatory cell recruitment to the joint cavity and nociception, were very similar at the tested doses. However, the administration of ATB-346 was not related to augmented gastric damage as was the case with the parent compound naproxen. Although naproxen and ATB-346 were not significantly different in terms of gastric MPO increase (after the oral administration of a single dose equivalent to 10 mg/kg naproxen), results from our laboratory show that the daily administration of naproxen at this dose during one week, leads to a significantly increase of gastric MPO by approximately 8-fold over the vehicle-treated control group, while this neutrophil marker remains unaltered in the ATB-346-treated animals (unpublished data).

Approximately 1–4% of the patients chronically treated with traditional NSAIDs suffer from gastric injuries, such as ulceration, bleeding, and/or obstruction [4], mainly mediated by the inhibition of gastroprotective prostaglandins, which leads to reduction of mucus and bicarbonate secretion, and thus decreasing the effectiveness of the mucosal pH gradient involed in the epithelium protection [5]. In this way, NSAIDs trigger an acute gastric inflammatory response, characterized by increased blood flow, plasma exudation and recruitment and activation of leukocytes to the mucosa [20].

One of the first attempts to overcome these adverse NSAID effects included the addition of nitric oxide (NO)-releasing moieties to the traditional NSAID structures, and in fact, these NO-releasing NSAIDs exhibited anti-inflammatory effects comparable to those of the parent compounds, in addition to reduced gastrointestinal and cardiovascular effects [21,22].

Over the last years, several studies have shown that H_2_S is a potent mediator of gastric mucosal protection, and the observed capacity of ATB-346 to minimize or prevent gastric lesions is attributable to its ability to release H_2_S. In fact, H_2_S donors can protect the gastric mucosa from NSAID-induced damage while, on the other hand, the inhibition of endogenous H_2_S synthesis results in significantly increased severity of NSAID-induced gastric damage [17,23].

H_2_S donors have been shown to reduce inflammation in several experimental situations, such as leukocyte-endothelium adherence inhibition in mesenteric venules, reduction of both paw [10] and articular edema [13], inhibition of lung allergy in mice [16] and myocardial ischemia-reperfusion injury [24]. Likewise, the slow-releasing H_2_S donor GYY4137, can reduce LPS-induced endotoxemia in mice [25], as well as the synthesis of pro-inflammatory mediators (such as TNF-α, IL-1β, IL-6, NO and PGE_2_) by LPS-stimulated RAW 264.7 macrophages in vitro [26].

Similarly to previous findings on the beneficial effects of ATB-346 in reducing paw inflammation in a model of adjuvant-induced arthritis [18], the present results show that both ATB-346 and naproxen have comparable good effectiveness in the carrageenan-induced synovitis. The injection of carrageenan into the knee joint induced significant increase in the number of neutrophils, in addition to mononuclear cells (monocytes/lymphocytes), and both naproxen and ATB-346 effectively reduced the migration of these cells to the joint cavity. Differently from the well known effects of the massive neutrophil migration to the joint cavity, the number of mononuclear cells found in the joint cavity are probably of no major relevance during the acute phase of the CGN-induced synovitis (i.e., 5 h after CGN injection). However, the significant reduction in the number of these cells that results from the treatment with either naproxen or ATB-346 will certainly affect the progression of the inflammatory situation (taking into account the pro-inflammatory mediators released by these cells at later periods) and will, in turn, favor the resolution of the inflammatory condition.

However, it is evident the lower activity of ATB-346 in comparison with naproxen to inhibit tactile alodynia and joint edema, as evaluated at the latest time-points post-dosing. It is likely that this reduced effectiveness of ATB-346 is due to the lower naproxen bioavailability that results from the oral administration of this compound which, according to the graph shown in Figure 5, means that the animals treated with ATB-346 were exposed to approximately 37% of the NSAID supplied by the oral administration of pure naproxen. On the other hand, equimolar doses of ATB-346 and naproxen showed no statistically different antiinflammatory effects in most of the measured parameters. Taken together, these observations could indicate that the low naproxen bioavailability that results from ATB-346 may be compensated by the antiinflammatory effects of the H_2_S released, although increases in serum total sulfide concentrations were not detected (data not shown). In fact, several previous studies have shown that the addition of a H_2_S-releasing moiety to traditional NSAID structures (which, for example, results in the mesalamine derivative ATB-429 or the diclofenac derivative ATB-337) increases the anti-inflammatory activity of the parent compounds [17,27], and show lower gastrointestinal toxicity [18].

Previously published studies have investigated the role of H_2_S in joint inflammation models and support its potential therapeutic use. For example, treatment of animals with sodium sulfide potently inhibited leukocyte infiltration, but rather unaffected joint pain secondary to kaolin/carrageenan-induced knee joint inflammation [12]. On the other hand, we reported that pre-treatment of rats with carrageenan-induced synovitis with the Lawesson's reagent (as a H_2_S donor) reduced not only leukocyte infiltration, but also edema and pain, in addition to decreased IL-1β production and increased constitutive nitric oxide synthase activity in the articular cavity [13]. In addition, diallyl sulfide (a garlic-derived H_2_S donor) was able to inhibit cyclooxygenase-2 expression and NF-κB activation in primary cultured synovial cells and chondrocytes stimulated with sodium urate crystals or IL-1β, in vitro [28]. In vitro experiments have also shown similar results regarding the beneficial effects of H_2_S in arthritis. For example, it was observed that the addition of NaHS to cultured fibroblasts extracted from patients with reumathoid arthritis reduced IL-6 production and inhibited MAPK activation [29] and that cysthationine gamma liase (CSE) is upregulated in cultured human articular chondrocytes and mesenchymal progenitor in the presence of pro-inflammatory cytokines [30], thus supporting the hypothesis that the increased synthesis of endogenous H_2_S could represent a novel mechanism of cytoprotection in human arthropaties.

Chronic NSAID treatment is the most common strategy to minimize joint inflammation and pain symptoms in arthritic patients, despite this can lead to higher risks of, not only gastrointestinal, but also cardiovascular complications. In this way, ATB-346 becomes a potentially attractive therapeutic alternative to traditional NSAIDs, considering that, in one hand, naproxen is one of the NSAIDs with less ability to trigger cardiovascular events [4,31] and, on the other hand, H_2_S exerts well documented protective effects on the cardiovascular system [8,11,32,33]. Indeed, Wallace et al. clearly demonstrated that while diclofenac or naproxen significantly elevated blood pressure in NO-deficient hypertensive rats, equimolar doses of the respective H_2_S-releasing derivatives ATB-337 or ATB-346 were devoid of significant effects [18].

## Conclusions

The present study shows that the presence of a H_2_S-releasing moiety in the ATB-346 structure does not impair the antiinflammatory activity of the parent compound, as evaluated in terms of inhibitory actions on joint pain, edema and inflammatory cell recruitment to the knee-joint of rats with carrageenan-induced synovitis. Instead, the antiinflammatory actions of the released H_2_S may compensate for the diminished naproxen bioavailability from ATB-346, as well as for the absence of deleterious gastric effects. As a whole, these characteristics make of ATB-346 an interesting therapeutic alternative to traditional naproxen to be potentially used in patients with arthritis.

## Abbreviations

ANOVA: Analysis of variance; ATB-346: The H_2_S-releasing naproxen derivative [2-(6-methoxy- napthalen-2-yl)-propionic acid 4-thiocarbamoyl-phenyl ester]; AUC: Area under the time vs. concentration curve; CGN: Carrageenan; COX: Cyclooxygenase; CSE: Cysthationine gamma liase; H2S: Hydrogen sulfide; HPLC: High-performance liquid chromatography; MS: Mass spectrometry; HPLC-MS-MS: High-performance liquid chromatography coupled to tandem electrospray mass spectrometry; i.art: Intra-articular; IL-1β: Interleukin 1β; IL-6: Interleukin 6; i.v: Intravenous; LPS: Gram-negative bacterial lipopolyssacharide; MAPK: Mitogen-activated protein kinases; MPO: Myeloperoxidase; NaHS: Sodium hydrosulfide; NO: Nitric oxide; NSAID: Non-steroidal antiinflammatory drug; PGE2: Prostaglandin E_2_; RA: Rheumatoid arthritis; p.o: Oral administration (per os); SEM: Standard error of the mean; TNF-α: Tumor necrosis factor α.

## Competing interests

JLW is a founder and shareholder of Antibe Therapeutics, Inc. All the other authors have no competing interests.

## Authors’ contributions

Experimental design: EEV, MAVP, LR, SAT, JLW, SKC & MNM. Execution of the experiments and data analysis: EEV, FPNM, KTS, MAVP, JF, CIZ, LR, GDN, SAT & MNM. Manuscript preparation and revision: EEV, FPNM, HHAF, JLW, SKC & MNM. All the authors read and approved the final manuscript.

## Aknowledgments

The authors would like to thank Mrs. Irene M. Gouvea and Mr. Mauro Sucupira for the technical assistance, and FAPESP, CNPq and CAPES for the financial support and fellowships.
